# Long-term outcomes of transcatheter atrial septal defect closure: a single-center retrospective study

**DOI:** 10.3389/fcvm.2024.1448967

**Published:** 2024-08-30

**Authors:** Lalita Honghiranrueng, Supaporn Roymanee, Kanjarut Wongwaitaweewong, Jirayut Jarutach, Rujira Buntharikpornpun

**Affiliations:** ^1^ Department of Pediatrics, Queen Sirikit National Institute of Child Health, Bangkok, Thailand; ^2^ Division of Pediatric Cardiology, Department of Pediatrics, Faculty of Medicine, Prince of Songkla University, Songkhla, Thailand

**Keywords:** patient outcomes, atrial septal defect, transcatheter, device closure, atrial fibrillation, pulmonary hypertension

## Abstract

**Background:**

Transcatheter atrial septal defect (ASD) closure is the primary approach for treating ASD secundum; however, data on long-term outcomes remain limited. This study aimed to elucidate the prevalence of adverse outcomes following transcatheter ASD closure in a diverse population.

**Methods:**

This retrospective cohort study was conducted at the Songklanagarind Hospital and included patients who underwent transcatheter ASD closure between January 2010 and August 2021.

**Results:**

The study included 277 patients who completed follow-up for at least 1 year, with varying ages: <25 years (31%), 25–40 years (19%), 40–60 years (34%), and >60 years (16%). The median follow-up duration was 37 months (interquartile range: 20, 61). The overall mortality rate was 1.8%, and no deaths were attributed to device-related complications. Hospitalization due to heart failure occurred in 0.7% of the cases. Most patients improved or stabilized based on the New York Heart Association functional class. Adverse outcomes included new-onset atrial fibrillation (prevalence: 2.7%) and pulmonary hypertension (prevalence: 0.6%). The resolution of pulmonary hypertension varied among age groups, with 100% resolution in patients <25 years. Multivariate analysis identified male sex, overweight, and history of stroke to be significantly associated with adverse outcomes after transcatheter ASD closure.

**Conclusion:**

Transcatheter ASD closure was safe and effective, with age not being a limiting factor for success. Male sex, being overweight, and a history of stroke were associated with adverse outcomes. These findings contribute to our understanding of the long-term outcomes following ASD closure.

## Introduction

Atrial septal defect (ASD) is the second most prevalent congenital heart anomaly, with an incidence of 164 cases per 100,000 live births, constituting 18% of all congenital heart defects (1). Secundum-type ASD accounts for 75% of cases (2). Although many patients remain asymptomatic or exhibit mild symptoms until adulthood, ASD can develop various complications including atrial fibrillation (AF), atrial flutter, pulmonary hypertension (PH), and heart failure (HF).

ASD closure is recommended in cases of significant left-to-right shunting, right-sided heart enlargement, or a history of paradoxical embolism (3). Transcatheter closure tends to be the preferred approach for secundum ASD, except when anatomical features preclude its feasibility; however, data on long-term outcomes remain limited. This study is designed to elucidate the prevalence of adverse outcomes following transcatheter ASD closure in a diverse population treated at our center.

## Materials and methods

### Study population

We conducted a retrospective cohort study at the Songklanagarind Hospital, a super-tertiary hospital in southern Thailand, of all patients who underwent transcatheter ASD closure between January 2010 and August 2021. This comprehensive investigation involved a review of the medical records, electrocardiograms, echocardiographic reports, and cardiac catheterization data of both inpatients and outpatients.

The inclusion criteria were patients with secundum-type ASD who underwent both left and right heart catheterization, met closure indications and had a minimum one-year follow-up period. Patients with associated heart diseases lost to follow-up within one year, and lacked essential cardiac catheterization data were excluded. The patients eligible for the study were classified into four groups based on their age at the time of the procedure: <25, 25–40, 40–60, and >60 years.

### Study design

The primary endpoints of the study were all-cause mortality and hospitalization owing to device-related complications. The secondary endpoint was cardiovascular morbidity related to AF or PH. Improvement in functional capacity was assessed at the last follow-up visit. Adverse outcomes were defined as complications leading to death, and new-onset AF or PH. Patients were followed up from the date of the procedure until the first recorded instance of mortality or until the end of the follow-up period, whichever occurred first. Follow-up information was gathered from inpatient and outpatient medical records, communication with the primary physicians of the patients, or phone interviews with the patients or their family members. The study was approved by the Faculty of Medicine, Prince of Songkla University's Ethics Committee (Approval No. REC.65-372-1-3, Approval date November 8, 2022). All the research steps were performed according to the ethical standards and principles under the 1975 Declaration of Helsinki and its later Amendments (2008). Written informed consent was obtained from the parents or the guardians of all patients included in the study.

### Data collection and definitions

Mortality was defined as all-cause mortality after transcatheter ASD closure. Baseline electrocardiograms and new-onset AF were evaluated. Pulmonary arterial hypertension (PAH) was defined according to the hemodynamic criteria outlined in the established guidelines (4): mean pulmonary artery pressure (mPAP) ≥20 mmHg, pulmonary vascular resistance (PVR) >2 Wood Units (WU) and mean pulmonary capillary wedge pressure ≤15 mmHg as documented during cardiac catheterization at baseline.

During follow-up, transthoracic echocardiography (TTE) was used to assess PH in cases where cardiac catheterization was not performed. The criteria for TTE assessment were right ventricular systolic pressure (RVSP) >40 mmHg or >25% of the systolic blood pressure. Additionally, the New York Heart Association (NYHA) functional class of the patients was evaluated at baseline and during the latest follow-up.

### Statistical analysis

Statistical analyses were performed using the R version 4.2.2 software (AT&T Bell Laboratories, Murray Hill, New Jersey). Continuous data are presented as mean ± standard deviation or median with interquartile range (IQR), as appropriate. Comparisons among age groups were done by using a one-way analysis of variance, the *F*-test for variables with a normal distribution, and the Kruskal–Wallis test for variables with an abnormal distribution. Categorical data are demonstrated as frequencies and percentages and were compared among age groups using Chi-square and Fisher's exact tests. Statistical significance was defined at *p* < 0.05.

## Results

Of the 407 patients who underwent transcatheter closure during the study period, 27 with associated congenital heart diseases, 41 lost to follow-up within one year, and 62 lacking essential cardiac catheterization data were excluded, resulting in a total exclusion rate of 31.9%.

A total of 277 patients were included in this study, wherein 85 (31%) were <25 years, 54 (19%) were aged 25–40 years, 94 (34%) were aged 40–60 years, and 44 (16%) were >60 years at the time of the procedure. Table 1 presents the demographic data of the study population categorized by age group. Significant differences were observed in most characteristics across the different age groups.

**Table 1:** Demographic data of the study population were categorized by age groups.

Variable	Overall (*n* = 277)	Age <25 (*n* = 85)	Age 25–40 (*n* = 54)	Age 40–60 (*n* = 94)	Age >60 (*n* = 44)	*P*-value
Female, *n* (%)	218 (78.7)	62 (72.9)	50 (92.6)	73 (77.7)	33 (75)	0.040
Age in years, median (IQR)	39 (20, 53)	8 (4, 16)	32 (29, 35)	48 (45, 53)	66 (62, 71)	<0.001
Weight in kg, median (IQR)	52.0 (43.0, 63.0)	22.0 (15.4, 47.0)	52.0 (49.0, 60.0)	59.5 (53.0, 70.8)	56.6 (49.9, 65.5)	<0.001
BMI in kg/m^2^, median (IQR)	21.3 (18, 24.7)	15.7 (14.1, 19.7)	21.0 (19.4, 23.8)	23.6 (20.7, 26.6)	24.0 (21.4, 26.4)	<0.001
Comorbidities, *n* (%)						
Smoking	15 (5.4)	1 (1.2)	1 (1.9)	7 (7.4)	6 (13.6)	0.011
Coronary artery disease	10 (3.6)	0 (0)	1 (1.9)	4 (4.3)	5 (11.4)	0.008
Diabetes mellitus	12 (4.3)	0 (0)	0 (0)	6 (6.4)	6 (13.6)	<0.001
Hypertension	11 (4.0)	1 (1.2)	1 (1.9)	26 (27.7)	21 (47.7)	<0.001
Atrial fibrillation	21 (7.6)	0 (0)	1 (1.9)	7 (7.4)	13 (29.5)	<0.001
Chronic lung disease	4 (2.4)	1 (1.2)	0 (0)	1 (1.1)	2 (4.5)	0.355
Stroke	4 (2.4)	0 (0)	1 (1.9)	1 (1.1)	2 (4.5)	0.152
Pulmonary hypertension	123 (44.4)	13 (15.3)	23 (42.6)	55 (58.5)	32 (72.7)	<0.001
Functional class, *n* (%)						<0.001
1	115 (41.5)	69 (81.2)	18 (33.3)	22 (23.4)	6 (13.6)	
2	142 (51.3)	16 (18.8)	34 (63.0)	63 (67.0)	29 (65.9)	
3	20 (7.2)	0 (0)	2 (3.7)	9 (9.6)	9 (20.5)	
Max ASD diameter, mm, mean (SD)	20.5 (5.4)	17.0 (4.9)	21.7 (5.8)	22.9 (4.4)	21.1 (4.3)	<0.001
ASD device/size ratio %, mean (SD)	121.6 (21.4)	116.3 (12.5)	126.0 (37.8)	121.9 (11.9)	125.1 (21.3)	<0.001
ASD occluder, *n*, (%)						0.622
Occlutech	239 (86.3)	72 (84.7)	47 (87.4)	83 (88.3)	37 (84.1)	
Amplatzer	31 (11.2)	12 (14.1)	6 (11.1)	9 (9.6)	4 (9.1)	
Cera	7 (2.5)	1 (1.2)	1 (1.85)	2 (2.1)	3 (6.8)	
Qp:Qs, median (IQR)	2.3 (1.8, 3.0)	2.0 (1.6, 2.8)	2.4 (1.8, 3.3)	2.4 (2.0, 3.1)	2.2 (1.8, 2.7)	0.004
mPAP mmHg, median (IQR)	21 (18, 26)	20 (16, 22)	20 (16, 25)	24 (20, 28)	26 (22, 29)	<0.001
PVR WU, median (IQR)	1.1 (0.7, 1.9)	1.1 (0.7, 1.5)	0.8 (0.5, 1.2)	1.3 (0.9, 2.1)	1.6 (1, 2.8)	<0.001
PVRi WU.m^2^, median (IQR)	1.6 (0.9, 2.6)	1.0 (0.7, 1.5)	1.2 (0.8, 2.0)	2.0 (1.3, 3.5)	2.6 (1.7, 4.3)	<0.001

IQR, interquartile range; BMI, body mass index; ASD, atrial septal defect; SD, standard deviation; mPAP, mean pulmonary artery pressure; PVR, pulmonary vascular resistance; WU, wood units; PVRi, pulmonary vascular resistance index.

We observed that of the 277 patients included, 218 (78.7%) were female, with a median age of 39 years (IQR: 20, 53). Patients <25 years exhibited a lower median body mass index (BMI) of 21.3 kg/m^2^ (IQR: 18, 24.7). Patients >60 years old manifested higher comorbidities, including smoking (13.6%), coronary artery disease (CAD) (11.4%), diabetes mellitus (13.6%), hypertension (47.7%), and AF (34%). The prevalence of PH increases with age. In addition, the NYHA functional class of patients tended to worsen with age.

Based on the cardiac catheterization data, the overall mean maximum diameter of the ASD was 20.5 ± 5.4 mm, and the mean ASD device-to-ASD diameter ratio was 121.6 ± 21.4%. The Occlutech ASD occluder (Occlutech GmbH, Jena, Germany) was the predominant device used in this study (86.3%). The mPAP, PVR, and pulmonary vascular resistance index (PVRi) showed increasing trends with age.

### Long-term outcomes among different age groups

In this study, 277 patients achieved complete ASD closure (Table 2). The median follow-up time was 37 months (IQR: 20–61 months). The overall all-cause mortality rate was 1.8%, and none of the deaths were related to the device. The hospitalization rate due to HF was 0.7%. The mortality incidence did not significantly differ among age groups (1.2%, 1.9%, 1.1%, and 4.5%; *p* = 0.415). The causes of death included severe infection, cancer-related causes, prior left ventricular (LV) dysfunction due to CAD, and progression of PAH. A 9-year-old boy with a tracheoesophageal fistula and bronchiectasis died of infected bronchiectasis 68 months after ASD closure. A 37-year-old female with severe PAH, mitral valve prolapses with mitral regurgitation (MR), and biventricular dysfunction died of biventricular HF 75 months after ASD closure. A 77-year-old female with CAD and severe MR, who had previously undergone prosthetic mechanical valve replacement, died of progressive LV dysfunction 69 months after ASD closure. A 59-year-old female with myelodysplastic syndrome and a 71-year-old male with diffuse large B-cell lymphoma died from cancer-related causes 72 and 15 months after ASD closure, respectively.

**Table 2:** Comparison of long-term outcomes between age groups.

Variable	Overall (*n* = 277)	Age <25 (*n* = 85)	Age 25–40 (*n* = 54)	Age 40–60 (*n* = 94)	Age >60 (*n* = 44)	*P*-value
Follow-up length in months, median (IQR)	37.0 (20, 61)	39.0 (18, 63)	36.5 (19, 68)	35.5 (19, 60)	41.0 (22, 57)	0.951
Long-term outcomes						
Death *n*, (%)	5 (1.8)	1 (1.2)	1 (1.9)	1 (1.1)	2 (4.5)	0.415
Heart failure hospitalization	2 (0.7)	0 (0)	1 (1.9)	0 (0)	1 (2.3)	0.291
New-onset AF *n*, (%)	7/171 (2.7)	0 (0)	2/53 (3.8)	4/87 (4.5)	1/31 (3.2)	0.189
Resolved AF *n*, (%)	4/21 (19.0)	0 (0)	1/1 (100)	1/7 (16.7)	2/13 (16.7)	0.272
New-onset PH *n*, (%)	1/154 (0.6)	0/72 (0)	0/31 (0)	1/39 (1.5)	0/12 (0)	0.532
Resolved PH *n*, (%)	95/123 (77.2)	13/13 (100)	19/23 (82.6)	40/55 (72.7)	23/32 (71.8)	0.122

IQR, interquartile range; AF, atrial fibrillation; PH, pulmonary hypertension.

The most frequent adverse outcome was new-onset AF, with a prevalence of 2.7%; however, none were detected in patients <25 years. The incidence of new-onset AF did not significantly differ among age groups (0%, 3.8%, 4.5%, and 3.2%; *p* = 0.189). Among the patients who manifested AF before transcatheter ASD closure, 17 of 21 (80%) had persistent AF during follow-up.

New-onset PH showed a prevalence of 0.6%, with no significant differences observed in its occurrence or resolution among the age groups. All patients <25 years of age achieved 100% PH resolution after transcatheter closure. However, the resolution rates in the other age groups, namely 25–40, 40–60, and >60 years, were 33.3%, 53.8%, and 46.7%, respectively.

**Figure 1:**
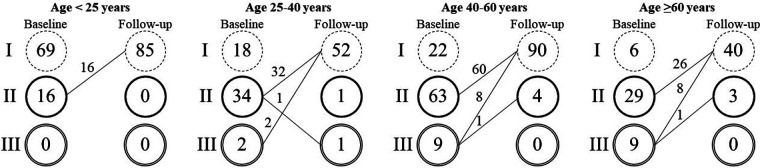
New York heart association functional capacity according to age.

Throughout the follow-up period, no cases of erosion, device embolization, thrombus formation, infective endocarditis, atrioventricular block, stroke, or complications associated with the intervention were observed. Most patients experienced improvement or stability in their NYHA functional class. Twenty patients (7.2%) showed improvement from NYHA functional class III to either class I (90%) or class II (10%) (Figure 1).

Several factors were associated with adverse outcomes, such as male sex, older age, higher BMI, hypertension, history of stroke, PH, larger ASD diameter, higher mPAP, and higher PVRi (Table 3). In the multivariate analysis, male sex, being overweight (BMI ≥25 kg/m^2^), and history of stroke were significantly associated with adverse outcomes (Table 4).

**Table 3:** Factors associated with adverse outcomes.

	Adverse outcomes (*n* = 13)	No adverse outcomes (*n* = 264)	*P*-value
Female n, (%)	6 (46.2)	212 (80.3)	0.003
Age in years, median (IQR)	52 (37, 59)	38.5 (19, 53)	0.025
BMI in kg/m^2^, median (IQR)	25.6 (23.7, 28.5)	21.1 (17.8, 24.4)	0.014
Comorbidities, *n* (%)			
Smoking	2 (15.4)	13 (4.9)	0.151
Coronary artery disease	1 (7.7)	9 (3.4)	0.387
Diabetes mellitus	1 (7.7)	11 (4.2)	0.543
Hypertension	6 (46.2)	43 (16.3)	0.015
Atrial fibrillation	1 (7.7)	20 (7.6)	0.988
Chronic lung disease	1 (7.7)	3 (1.1)	0.176
Stroke	2 (15.4)	2 (0.8)	0.012
Pulmonary hypertension	10 (76.9)	113 (42.8)	0.033
Maximum ASD diameter in mm, mean (SD)	23.4 (6.7)	20.4 (5.3)	0.048
ASD device/size ratio %, mean (SD)	120.8 (17.4)	121.6 (21.6)	0.886
Qp:Qs, median (IQR)	2.2 (1.9, 3.3)	2.3 (1.8, 3.0)	0.886
mPAP mmHg, median (IQR)	26 (23, 33)	21 (18, 26)	0.008
PVR WU, median (IQR)	1.5 (1.1, 2.1)	1.1 (0.7, 1.9)	0.075
PVRi WU.m^2^, median (IQR)	2.4 (2.0, 3.0)	1.5 (0.91, 2.6)	0.006

IQR, interquartile range; BMI, body mass index; ASD, atrial septal defect; SD, standard deviation; mPAP, mean pulmonary artery pressure; PVR, pulmonary vascular resistance; WU, wood units; PVRi, pulmonary vascular resistance index.

**Table 4:** Multivariate analysis of factors associated with adverse outcomes.

Factor	Odds ratio	95% CI	*P*-value
Female	0.14	(0.032–0.571)	0.007
Age >25 years	1.40	(0.123–15.952)	0.785
Overweight (BMI ≥25 kg/m^2^)	6.75	(1.460–31.224)	0.014
Hypertension	1.60	(0.400–6.371)	0.508
Stroke	26.86	(1.690–427.082)	0.020
Pulmonary hypertension	4.56	(0.835–24.887)	0.080
ASD size >20 mm	0.92	(0.186–4.541)	0.918
mPAP ≥20 mmHg	2.42	(0.254–23.025)	0.443
PVR >2 Wood unit	2.45	(0.005–1,260.589)	0.778
PVRi ≥4 Wood unit × m^2^	3.91	(0.007–2,064.329)	0.670

CI, confidence interval; BMI, body mass index; ASD, atrial septal defect; mPAP, mean pulmonary artery pressure; PVR, pulmonary vascular resistance; PVRi, pulmonary vascular resistance index.

## Discussion

Previous studies have consistently demonstrated that transcatheter ASD closure reduces mortality, improves functional capacity, and has low morbidity post-procedure (5–8). Our study was conducted at a single referral center in southern Thailand from 2010 to 2021, reaffirming these findings by reporting that all-cause mortality was 1.8%, which aligns with previous studies ranging from 0.47%–2.4% (9–11), with no deaths attributed to device-related complications.

Arrhythmias, particularly AF, are common in patients with ASD, especially in older individuals. In our study, the incidence of new-onset AF post-procedure (2.7%) was lower than that reported in other studies (9.2%–14.9%) (12, 13). This discrepancy may be due to our use of only simple ECGs before the procedure and during follow-up, which are less optimal for diagnosing paroxysmal AFib. Consequently, the true incidence of pre-existing and/or new-onset AFib may be underestimated. Further investigation is needed to accurately determine the incidence, especially in higher-risk patients over 40 years of age and elderly patients. Among the patients who manifested AF before transcatheter ASD closure, 80% had persistent AF during follow-up. This finding emphasizes that transcatheter ASD closure was not capable of restoring sinus rhythm in patients who presented with AF beforehand, consistent with a study by Vecht et al. (14). There were no significant differences in the occurrence of new-onset AF between the different age groups. Furthermore, it is important to highlight that we did not observe new-onset stroke following transcatheter closure in our study cohort. This indicated that the procedure did not increase the risk of stroke in patients undergoing ASD closure.

In our study, we encountered only one patient who developed new-onset PH following the closure of ASD. This patient had increased RVSP from 35 to 41 mmHg, which falls under the category of intermediate probability of PH (4). Further evaluation was scheduled through a follow-up echocardiogram since cardiac catheterization was not performed to assess the progression and severity of PH. This approach may introduce errors due to the use of two different methods for evaluating PH. Consistent with a study by D'Alto et al. (15), we observed that patients who manifested PH after defect closure often had a high baseline PVR and PVRi. In our study, 28 patients had persistent PH, with eight patients having PVR values >5 WU at the time of the procedure. We observed that the 28 patients who had persistent PH had a median PVRi of 4.0 WU.m^2^, which aligns with a report by Jarutach et al. that stated that a pre-repair PVRi of <4 WU.m^2^ was associated with better survival than a higher PVRi (16).

Most patients showed improvement in the NYHA functional class except for one patient with severe PAH whose functional class deteriorated from II to III after 75 months of follow-up. This patient initially presented with a large ASD, MVP with MR, severe PAH, fair biventricular function, and NYHA functional class IV, which improved to class II after receiving anti-HF medications and pulmonary vasodilator drugs (only sildenafil was administered owing to economic strain). Data at the time of the procedure revealed an ASD size of 37 × 35 mm, systolic pulmonary artery pressure (PAP) of 80% of the systemic pressure, mPAP of 49 mmHg, Qp:Qs ratio of 3.3, PVR of 6.7 WU, Rp: Rs of 0.19, and LVEDP of 10 mmHg. In 2012, the year of ASD closure, no fenestrated ASD occluder was available at our center, so the patient's ASD was closed by a 39 mm Occlutech ASD occluder. Immediate follow-up was uneventful and the patient clinically improved; however, PAH persisted, and sildenafil was still administered at the maximal dosage. Five-year post-procedure, the functional class of the patient changed from II to III, and the hemodynamic data showed that PAP was 77% of the systemic pressure, mPAP 72 mmHg, PVR 19.9 WU, and Rp: Rs of 0.36, which explains the progression of PAH and biventricular failure. We emphasize that PH is not a benign condition. This was particularly true in patients with PVRi >4 WU.m^2^ and PVR >5 WU. According to the current European Society of Cardiology guidelines, ASD closure is not recommended for patients with PAH, and a PVR ≥5 WU, despite targeted PAH treatment. However, if PVR falls below 5 WU after targeted PAH treatment and the Qp:Qs >1.5, closure of the defect with a fenestrated ASD may be considered (3). A previous study suggests that if the PVR is <5–7 WU, the treat-and-repair strategy is still effective (17).

In our multivariate analysis, we found that patients >25 years and mPAP ≥20 mmHg were not associated with adverse outcomes in patients who underwent transcatheter ASD closure. However, we identified three variables that were significantly associated with poor outcomes, which were male sex, being overweight, and having a history of stroke. Stroke is the foremost cause of mortality and morbidity, particularly when combined with AF (18).

ASD closure changes the natural course of the disease, wherein it reduces mortality and improves the functional capacity of the patients after the procedure. However, patients with ASD still have a higher mortality rate than the general population (19). Long-term morbidity due to arrhythmia, HF, PH, and stroke still warrant long-term monitoring (20). AF remains a major issue to explore and whether ASD closure will lessen or worsen its occurrence (21). In this study, the sample size of patients with adverse outcomes was relatively small. Therefore, further research with a larger cohort is necessary to strengthen the evidence and provide more robust conclusions regarding the predictors of long-term complications including arrhythmia, HF, PH, erosion, stroke, thrombotic complication, infective endocarditis, incomplete neo-endothelialization and the interrelationship between those problems that affect survival outcomes in patients undergoing transcatheter ASD closure.

## Limitations

This study had several limitations. Our center is a referral center for 14 provinces in southern Thailand. Most patients experience economic strain and frequent changes in cell phone numbers, resulting in a lack of continuous follow-up. The follow-up duration may not have been sufficient to capture all long-term outcomes. Furthermore, the absence of a control group, particularly age- and sex-matched controls, limited the ability to make direct comparisons and draw definitive conclusions. The baseline features of our population and the presence of comorbidities may differ significantly because of the wide range of age of the patients.

Data assessment relied on information extracted from medical records, and functional capacity evaluation was based on the subjective impressions of the patients using the NYHA functional class. Cardiopulmonary exercise testing, which provides a more objective measure of functional capacity, was not performed in this study. However, it should be noted that cardiopulmonary exercise testing may be challenging to perform in certain patient populations, such as in children and the elderly.

While efforts were made to ensure regular follow-up of patients, 31.9% were excluded. Some limitations may exist owing to patients being lost to follow-up. Multicenter or larger prospective studies with control groups are necessary to validate the long-term outcomes of transcatheter ASD closure.

## Conclusion

In conclusion, our study affirms the safety and effectiveness of transcatheter ASD closure in patients of all ages. We identified male sex, being overweight, and stroke as risk factors associated with adverse outcomes after the procedure. Further research is needed to corroborate these findings and explore the underlying mechanisms in more detail.

## Data Availability

The raw data supporting the conclusions of this article will be made available by the authors, without undue reservation.
